# PCB118 Induces Inflammation of Islet Beta Cells via Activating ROS-NLRP3 Inflammasome Signaling

**DOI:** 10.1155/2021/5522578

**Published:** 2021-05-17

**Authors:** Chunxia Jiang, Yuping Wang, Man Guo, Yang Long, Jiao Chen, Fang Fan, Shi Tang, Yong Xu

**Affiliations:** ^1^Department of Endocrinology and Metabolism, The Affiliated Hospital of Southwest Medical University, Sichuan Luzhou 646000, China; ^2^Department of Vascular and Breast Surgery, Hospital (T.C.M) Affiliated to Southwest Medical University, Sichuan, Luzhou 646000, China; ^3^Cardiovascular and Metabolic Diseases Key Laboratory of Luzhou, Affiliated Hospital of Southwest Medical University, Luzhou, Sichuan 646000, China; ^4^Sichuan Clinical Research Center for Nephropathy, Luzhou, Sichuan 646000, China; ^5^Department of Endocrinology, The Third Hospital of Mianyang, Sichuan 621000, China; ^6^Department of Endocrinology, The First People's Hospital of Neijiang, Sichuan 641000, China; ^7^Endocrinology Department, The Affiliated Hospital of Nuclear Industry 416 Hospital, Chengdu, Sichuan 610000, China

## Abstract

**Background:**

Diabetes mellitus is a clinical syndrome caused by genetic and environmental factors. Growing evidence suggests that exposure to environmental endocrine disruptors and activation of NLRP3 inflammasome signaling play a vital role in diabetes. However, it is unclear how PCB118, a common environmental endocrine disruptor, contributes to the incidence of diabetes, and its specific mechanism of action is unknown. In this study, we explored whether ROS-induced NLRP3 inflammasome priming and activation were related to PCB118 exposure in mouse islet *β*-TC-6 cells and the mechanisms of diabetes.

**Methods:**

Mouse islet *β*-TC-6 cells were cultured with PCB118 as a stimulating factor and ROS inhibitor N-acetyl cysteine (NAC) as an intervention. Cellular toxicity due to PCB118 was detected using the Cell Counting Kit-8; ROS was measured using DCFH-DA; the expressions of NLRP3, procaspase-1, caspase-1, pro-IL-1*β*, and IL-1*β* protein were detected by western blot; and IL-6, IL-18, and C-C chemokine ligand 2 (CCL-2) were measured by ELISA.

**Results:**

PCB118 caused significant toxicity to the cells when the stimulation concentration was equal to or greater than 80 nmol/L at 72 hours (*p* < 0.05) and increased the levels of ROS, NLRP3, caspase-1, IL-1*β*, IL-6, IL-18, and CCL-2 (*p* < 0.05); the expressions of procaspase-1 and pro-IL-1*β* were downregulated in a dose-dependent manner after PCB118 exposure (*p* < 0.05), which was prevented by pretreatment with NAC (*p* < 0.05).

**Conclusions:**

PCB118 can activate NLRP3 inflammasome signaling in islet beta cells via the oxidative stress pathway and cause inflammation in islet beta cells. It suggests that environmental endocrine disruptors play an important role in the inflammation of islet beta cells and may contribute to the development of diabetes through NLRP3 inflammatory signaling.

## 1. Introduction

Diabetes is the second most common chronic noncommunicable disease. In 2019, the International Diabetes Federation (IDF) reported that 1 in 11 adults (20-79 years) have diabetes (463 million people), 374 million people have impaired glucose tolerance, and 232 million people are undiagnosed [1]. The acute and chronic complications of diabetes seriously affect the quality of life and lifespan of patients. However, the etiology and pathogenesis of diabetes are complex. Studies have shown that diabetes is caused by genetic and environmental factors, including environmental pollution [2–4].

Polychlorinated biphenyls (PCBs) are a class of persistent organic pollutants (POPs), which are one of the most important environmental pollutants. They are artificially synthesized organic compounds that persist in the environment, accumulate through the food web, and cause adverse effects on the human body and the environment. Numerous studies have shown that PCBs exposure is associated with diabetes, gestational diabetes (GDM), hypertension, and heart failure [5–9]. 2,3′,4,4′,5-Pentachlorobiphenyl (PCB 118), a coplanar PCB and a common environmental endocrine disruptor, is lipophilic, stable, resistant to degradation, and closely associated with the occurrence of diabetes [6, 10]. However, PCB118's specific mechanism of action and how it may lead to diabetes are still unknown.

A central component of diabetes is the occurrence of a long-term low-level inflammatory response. Oxidative stress plays a significant role in diabetes and its complications [11–14]. The NLRP3 inflammasome plays an important role in the innate immune-mediated inflammatory response and is activated by intracellular and extracellular signaling molecules in several ways, including the production of reactive oxygen species (ROS). NLRP3 inflammasome activation results in the activation of caspase-1, which cleaves the cytokines prointerleukin-1*β* (pro-IL-1*β*) and prointerleukin-18 (pro-IL-18) into their mature and biologically active forms (IL-1*β* and IL-18), promoting an inflammatory reaction [15–17].

Studies have shown that NLRP3 inflammasome activation plays an important role in the development of diabetes and diabetic complications [18, 19]. Some studies have found that oral treatment with aroclor 1254, a PCB mixture, impairs glucose tolerance and results in the failure of *β*-cells to make sufficient levels of insulin in mice [20]. Exposure to PCB126 can result in deviant development of the pancreatic islet [21], and exposure to PCBs can induce high levels of ROS in islet cells [22]. PCB29-pQ induced inflammatory cytokines, such as tumor necrosis factor (TNF-*α*), interleukin-6 (IL-6), and IL-1*β*; the overproduction of ROS by PCB29-pQ played significant roles in the process [23]. Additionally, our previous study found that high glucose and lipopolysaccharide can prime the NLRP3 inflammasome via the ROS/TXNIP pathway in mesangial cells, and PCB118 can promote inflammation in endothelial cells [24, 25]. Thus far, the role of PCB118 in priming ROS-induced NLRP3 inflammasome activation in mouse islet beta cells is controversial. We therefore designed our investigations based on the hypothesis that exposure to PCB118 could prime and activate the NLRP3 inflammasome via the ROS pathway in mouse islet *β*-TC-6 cells.

## 2. Materials and Methods

### 2.1. Materials

Mouse islet *β*-TC-6 cells were purchased from TONGPAI (Shanghai) Biotechnology Co. Ltd. PCB118 was purchased from AccuStandard Inc. (New Haven, CT, USA). Cell Counting Kit-8 (CCK8), ROS detection kit, and DMSO were purchased from Beyotime Institute of Biotechnology (Shanghai, China). Specific antibodies against NLRP3, caspase-1, and fetal bovine serum were purchased from Cell Signaling Technology (CST, Boston, MA, USA). Anti-caspase-1 antibody (EPR4321) and anti-IL-1 *β* antibody were purchased from Abcam (USA). GAPDH polyclonal antibody was purchased from BioWorld (USA). ELISA kits for IL-6, IL-18, and CCL-2 were purchased from Cheng Lin Biological (Beijing, China).

### 2.2. Cell Culture and Cell Viability

Mouse islet *β*-TC-6 cells were seeded in 96-well culture plates and cultured with various concentrations of PCB118 (5, 10, 20, 40, 80, 160, or 320 nmol/L), DMSO, and 15% fetal bovine serum for 48 hours or 72 hours. The Cell Counting Kit-8 (CCK8) was used to detect cell viability. Based on the results of the cell viability assay after exposure to PCB118, cells were grown as monolayers at a density of 5 × 10^4^ cells/cm^2^ at 37°C in humidified air containing 5% CO_2_ and randomly divided into the following groups and treatments: the DMSO group (solvent control group, the concentration of DMSO was equal to the concentration of the DMSO present in the PCB118 (20 nmol/L)), the PCB118 groups (5, 10, and 20 nmol/L), and the PCB118 (20 nmol/L)+NAC (N-acetyl cysteine, 10 *μ*mol/L) group.

### 2.3. ELISA

Cells were cultured as detailed above for 48 hours, and the levels of IL-6, IL-18, and CCL-2 in the cell supernatants were measured using ELISA kits according to the manufacturer's instructions.

### 2.4. Reactive Oxygen Species (ROS) Evaluation

Mouse islet *β*-TC-6 cells were grown in 24-well culture plates in the above-mentioned conditions for 48 hours and were then incubated in serum-free media containing DCFH-DA (10 *μ*mol/L) for 30 minutes. The levels of intracellular ROS were evaluated using the ROS detection kit. The conversion of DCFH-DA was measured using a spectrofluorometer with excitation at 488 nm and emission at 525 nm.

### 2.5. Western Blotting

Cells were grown in 6-well culture plates in the conditions mentioned above for 48 hours. Next, the cells were lysed with radioimmunoprecipitation assay (RIPA) lysis and extraction buffer containing complete protease inhibitor mixture, and lysates of cells were resolved by SDS-PAGE and transferred to PVDF membranes by electroblotting. Immunoblotting was performed using specific antibodies against NLRP3 (rabbit, 110 kDa, 1 : 800; CST, USA), procaspase-1 (rabbit, 48 kDa, 1 : 2000; Abcam, USA), caspase-1 (rabbit, 25 kDa, 1 : 500; CST, USA), pro-IL-1*β* (rabbit, 31 kDa, 1 : 1500; Abcam, USA), IL-1*β* (rabbit, 17 kDa, 1 : 800; Abcam, USA), and GAPDH (37 kDa, 1 : 2000; BioWorld, USA). Bands were visualized using HRP-conjugated secondary antibodies (anti-rabbit and anti-mouse, 1 : 2000, Beyotime, China) and quantified using Quantity One software (Bio-Rad Laboratories, Hercules, USA).

### 2.6. Statistical Analysis

All experimental data were obtained from three independent experiments, and a representative example is shown. Data are expressed as the mean ± SD (*x* ± *s*) and were analyzed using the SPSS 17.0 statistical package. One-way analysis of variance and LSD-*t* were used to analyze all experimental data. The homogeneity test of variance was run for every group, and *p* < 0.05 was considered to be statistically significant.

## 3. Results

### 3.1. The Effect of PCB118 on Cell Viability in Mouse Islet *β*-TC-6 Cells

**Figure 1:**
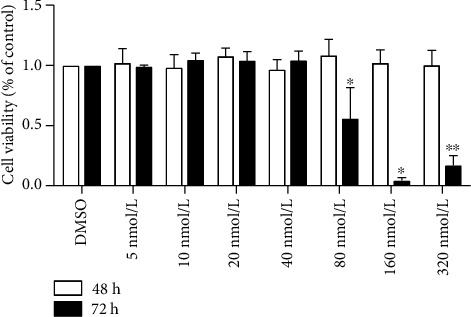
The effect of PCB118 on cell viability in mouse islet *β*-TC-6 cells. Cells were cultured with various concentrations of PCB118 (5, 10, 20, 40, 80, 160, or 320 nmol/L) for 48 hours or 72 hours. The Cell Counting Kit-8 (CCK8) was used to detect cell viability. PCB118 had no effect on cell viability at concentrations from 5 to 320 nmol/L after 48 hours, but after exposure for 72 hours at a concentration equal to or greater than 80 nmol/L, PCB118 decreased the cell viability significantly. ^∗^*p* < 0.05. ^∗∗^*p* > 0.05 vs. PCB118 160 nmol/L.

In order to detect the toxic effect of PCB118 on cells, we tested whether PCB118 exposure affects cell viability in mouse islet *β*-TC-6 cells using the Cell Counting Kit-8 (CCK8). The results showed that PCB118 had no effect on cell viability at concentrations from 5 to 320 nmol/L after 48 hours (*p* > 0.05), but after exposure for 72 hours at a concentration equal to or greater than 80 nmol/L, the cell viability decreased significantly; the administration of 80, 160, and 320 nmol/L PCB118 decreased cell viability by approximately 44%, 96%, or 83%, respectively (*p* < 0.05), while there was no difference between 160 nmol/L and 320 nmol/L (*p* > 0.05, Figure 1). Therefore, we chose concentrations of PCB118 that had no effect on cell viability for further study, and cells were divided into the following groups for subsequent experiments: the DMSO (solvent control) group and the PCB118 groups (5 nmol/L, 10 nmol/L, 20 nmol/L).

### 3.2. PCB118 Activates Cellular Inflammation in Mouse Islet *β*-TC-6 Cells

**Figure 2:**
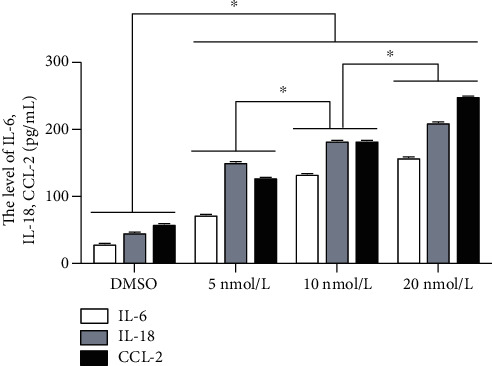
The levels of IL-6, IL-18, and CCL-2 in the cell supernatants activated by PCB118. Cells were cultured with PCB118 (5, 10, or 20 nmol/L) for 48 hours, and the levels of IL-6, IL-18, and CCL-2 in the cell supernatants were measured using ELISA kits. The production of IL-6, IL-18, and CCL-2 was significantly increased in the PCB118 groups in a dose-dependent manner. ^∗^*p* < 0.05.

Diabetes is a metabolic disorder characterized by hyperglycemia and insulin resistance, and oxidative stress and inflammation play key roles in the onset and development of this condition [12, 26]. Therefore, we assessed the levels of inflammatory mediators, IL-6, IL-18, and CCL-2, in the cell culture supernatants via ELISA. The results showed that compared to the DMSO group, the production of IL-6, IL-18, and CCL-2 was significantly increased in the PCB118 groups in a dose-dependent manner (*p* < 0.05, Figure 2). These results suggest that PCB118 can stimulate islet beta cells to produce inflammatory factors and cause inflammation.

### 3.3. PCB118-Induced Inflammation Is Dependent upon NLRP3 Inflammasome Activation

**Figure 3:**
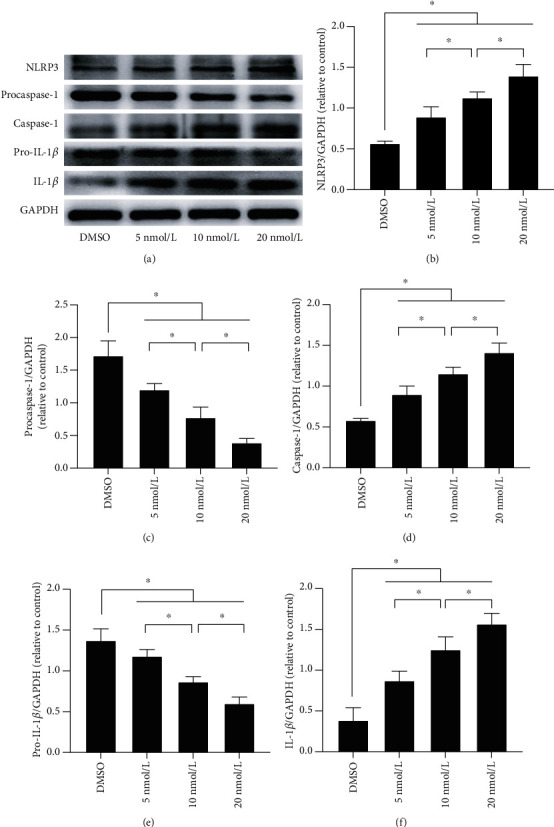
PCB118 activated NLRP3 inflammasome signaling in mouse islet *β*-TC-6 cells. The expression of NLRP3 was increased in the PCB118-treated cells, procaspase-1 and pro-IL-1*β* were cleaved into active caspase-1 and IL-1*β*, and the expression of caspase-1 and IL-1*β* increased significantly in the PCB118 groups in a dose-dependent manner (a–f). ^∗^*p* < 0.05.

As previously mentioned, the NLRP3 inflammasome plays an important role in the innate immune-mediated inflammatory response. Therefore, we measured the levels of NLRP3 inflammasome-related proteins after PCB118 exposure by western blotting to determine whether the increase in the expression of inflammatory factors caused by PCB118 was related to activation of the NLRP3 inflammasome. As expected, compared to the DMSO group, the expression of NLRP3 was increased in the PCB118-treated cells, procaspase-1 and pro-IL-1*β* were cleaved into active caspase-1 and IL-1*β*, and the expression of caspase-1 and IL-1*β* increased significantly in the PCB118 groups in a dose-dependent manner (*p* < 0.05, Figures 3(a)–3(f)), especially for the 20 nmol/L PCB118 group.

### 3.4. ROS Mediates PCB118-Induced NLRP3 Inflammasome Activation in Mouse Islet *β*-TC-6 Cells

**Figure 4:**
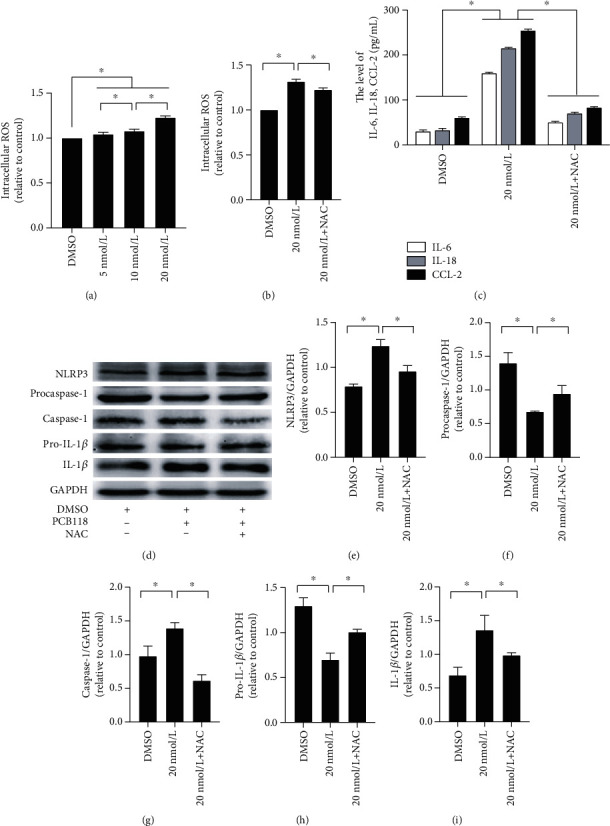
ROS mediates PCB118-induced NLRP3 inflammasome activation in mouse islet *β*-TC-6 cells. The production of ROS in the PCB118-treated cells was significantly increased (a). Cells were pretreated with 10 *μ*mol/L ROS inhibitor NAC and then exposed to 20 nmol/L PCB118 for 48 hours. NAC significantly inhibited intracellular ROS production (b) and the production of IL-6, IL-18, and CCL-2 (c). The expression of NLRP3, caspase-1, and IL-1*β* also decreased significantly, while the expression of procaspase-1 and pro-IL-1*β* recovered to a certain degree (d–i). ^∗^*p* < 0.05.

In this study, DCFH-DA assays were used to examine the production of whole ROS. Compared to the DMSO group, the production of ROS in the PCB118-treated cells was significantly increased in a dose-dependent manner (*p* < 0.05, Figure 4(a)). To further verify that the activation of the NLRP3 inflammasome is associated with increased ROS production, cells were pretreated with 10 *μ*mol/L ROS inhibitor NAC and then exposed to 20 nmol/L PCB118 for 48 hours. We found that NAC significantly inhibited intracellular ROS production (*p* < 0.05, Figure 4(b)) and the production of IL-6, IL-18, and CCL-2 (*p* < 0.05, Figure 4(c)). The expression of NLRP3, caspase-1, and IL-1*β* also decreased significantly (*p* < 0.05), while the expression of procaspase-1 and pro-IL-1*β* recovered to a certain degree (*p* < 0.05, Figures 4(d)–4(i)). These results indicate that the activation of NLRP3 inflammasome is related to the increase in ROS production, PCB118 activates the cellular inflammatory response by activating the ROS-NLRP3 inflammasome signaling pathway, and NAC can alleviate this response to a certain extent.

## 4. Discussion

Growing epidemiological and experimental evidence has shown that exposure to endocrine disrupting chemicals, including PCBs, is harmful to the endocrine system and may cause diabetes. A long-term inflammatory response, oxidative stress, and release of ROS, as well as activation of subsequent NLRP3 inflammasome signaling, are significantly involved in the pathogenesis of diabetes [19, 24]. Studies have shown that PCB118 can increase the production of ROS, induce oxidative stress, and activate the NLRP3 inflammasome [25, 27, 28]. Our study was conducted to explore the potential role and mechanisms of PCB118 and ROS-NLRP3 inflammasome signaling in the inflammation of islet beta cells and diabetes. The results demonstrated that prolonged exposure to PCB118 at high doses may result in decreased islet cell viability. Moreover, we found that PCB118 exposure induces increased production of inflammatory factors and excessive intracellular ROS, which participates in oxidative stress. Furthermore, we found that PCB118-induced inflammation is dependent upon ROS-mediated NLRP3 inflammasome priming and activation.

One study found that diabetes, hypertension, obesity, and high serum lipids were strongly associated with certain PCBs [29]. In another study, the cardiovascular mortality dose-dependent association for dietary PCB exposure had a hazard ratio (HR) of 1.31 (CI 95%: 1.08 to 1.57; *p* trend 0.005) [30]. Han et al. [3] found significantly positive associations between PCBs (including PCB-77, 81, 105, 114, 118, 123, 126, 156, 157, 167, 169, 189, 28, 52, 101, 138, 153, 180, 209, 202, 205, and 208) and the risk of type 2 diabetes, and PCBs showed a linear dose-response relationship with diabetes. These findings indicate that exposure to PCBs may be a diabetogenic factor. In further support of this hypothesis, *in vivo* PCB126 exposure increased free radical generation and modified the expression of proteins related to oxidative stress in islets of Langerhans, which is indicative of early *β*-cell failure [31]. PCBs exposure may directly result in failure of the *β*-cell mass to make sufficient levels of insulin [20]. PCBs also induce high levels of ROS and oxidative stress, suggesting that they may potentially induce damage in islet cells [22]. In our current study, we discovered that when the concentration of PCB118 was greater than 80 nmol/L for 72 hours, PCB118 decreased islet beta cell viability, indicating that prolonged exposure to PCB118 at high doses may have a toxic effect on islet beta cells. Additionally, PCB118 increased the production of inflammatory factors (IL-6, IL-18, and CCL-2) that may damage islet cells, possibly leading to the occurrence and development of diabetes.

The Nod-like receptor protein 3 (NLRP3) inflammasome is a pattern recognition receptor located in the cytoplasm. It is composed of NLRP3, apoptosis-associated speck-like protein (ASC), and aspartate-specific caspase-1 combined to mediate innate immunity and adaptive immunity [32]. NLRP3 inflammasome activation requires the following steps: pattern recognition receptors recognize pathogen-related molecular patterns (PAMPs, such as viruses or bacteria) or damage-associated molecular patterns (DAMPs, such as hyperglycemia or hyperlipidemia) and activate NF-*κ*B signaling. This promotes the transcription of NLRP3, ASC, and procaspase-1 and then induces their assembly and activation through ROS, lysosome destruction, K+ channels, and other methods. Finally, cleavage of procaspase-1 and splicing of pro-IL-1*β* and pro-IL-18 occur to promote the maturation and secretion of IL-1*β* and IL-18, ultimately triggering an inflammatory cascade reaction [33–35]. Studies have shown that high glucose induces ROS production, activates the NLRP3 inflammasome, and promotes cytokine secretion and that an ROS inhibitor can reduce the NLRP3 inflammasome activation and cytokine secretion [24, 36, 37]. In our study, we found that PCB118 exposure induces increased production of excessive intracellular ROS and positively regulates NLRP3 inflammasome signaling via the oxidative stress pathway, promotes the maturation and secretion of IL-1*β* and IL-18, and ultimately triggers an inflammatory cascade reaction. This may eventually lead to the occurrence and development of diabetes.

## 5. Conclusions

In conclusion, we have demonstrated that PCB118 plays an important role in the inflammation of islet beta cells and possible association with diabetes. Decreased islet beta cell viability occurred upon exposure to a high concentration of PCB118. Additionally, we found that PCB118-induced inflammation is dependent upon ROS-NLRP3 inflammasome priming and activation, which may be a potential mechanism of diabetes pathogenesis. These results provide further research directions for the prevention and treatment of diabetes.

## Data Availability

The data used to support the findings of this study are included within the article and available from the corresponding author upon request.
